# Communication about Children's Clinical Trials as Observed and Experienced: Qualitative Study of Parents and Practitioners

**DOI:** 10.1371/journal.pone.0021604

**Published:** 2011-07-12

**Authors:** Valerie Shilling, Paula R. Williamson, Helen Hickey, Emma Sowden, Michael W. Beresford, Rosalind L. Smyth, Bridget Young

**Affiliations:** 1 Institute of Psychology, Health and Society, University of Liverpool, Liverpool, United Kingdom; 2 Department of Biostatistics, Institute of Translational Medicine, University of Liverpool, Liverpool, United Kingdom; 3 Medicines for Children Research Network Clinical Trials Unit, Alder Hey Children's NHS Foundation Trust, Liverpool, United Kingdom; 4 Department of Women's and Children's Medicine, Institute of Translational Medicine, University of Liverpool, Alder Hey Children's NHS Foundation Trust, Liverpool, United Kingdom; 5 University of Liverpool, Alder Hey Children's NHS Foundation Trust, Liverpool, United Kingdom; IUMSP CHUV/University of Lausanne, Switzerland

## Abstract

**Background:**

Recruiting children to clinical trials is perceived to be challenging. To identify ways to optimise recruitment and its conduct, we compared how parents and practitioners described their experiences of recruitment to clinical trials.

**Methods and Findings:**

This qualitative study ran alongside four children's clinical trials in 11 UK research sites. It compared analyses of semi-structured interviews with analyses of audio-recordings of practitioner-family dialogue during trial recruitment discussions. Parents from 59 families were interviewed; 41 had participated in audio-recorded recruitment discussions. 31 practitioners were interviewed. Parents said little in the recruitment discussions contributing a median 16% of the total dialogue and asking a median of one question. Despite this, parents reported a positive experience of the trial approach describing a sense of comfort and safety. Even if they declined or if the discussion took place at a difficult time, parents understood the need to approach them and spoke of the value of research. Some parents viewed participation as an ‘exciting’ opportunity. By contrast, practitioners often worried that approaching families about research burdened families. Some practitioners implied that recruiting to clinical trials was something which they found aversive. Many were also concerned about the amount of information they had to provide and believed this overwhelmed families. Whilst some practitioners thought the trial information leaflets were of little use to families, parents reported that they used and valued the leaflets. However, both parties agreed that the leaflets were too long and wanted them to be more reader-friendly.

**Conclusions:**

Parents were more positive about being approached to enter their child into a clinical trial than practitioners anticipated. The concerns of some practitioners, that parents would be overburdened, were unfounded. Educating practitioners about how families perceive clinical trials and providing them with ‘moral’ support in approaching families may benefit paediatric research and, ultimately, patients.

## Introduction

Clinical trials are essential for evaluating healthcare and improving treatments for all patient populations. However, most paediatric specialties have historically had low levels of clinical trial activity [1], [2]. Children have been perceived as a vulnerable population [3] whose involvement in research should be minimised in order to protect them and clinical trials in paediatrics have been regarded as economically unviable, resulting in children being excluded from the benefits of evidence-based medicine [4]. Gradual realization of the inequities and risks this exclusion posed to children's health has led to international policy changes to augment paediatric clinical trial activity [2], [5], [6], [7]. While these changes have increased the number of clinical trials being conducted with children, considerable challenges remain. Poor accrual is one of the principal reasons for clinical trial failure [8]. Children's trials may be especially at risk of accrual problems due, for example, to the relatively small numbers of paediatric patients with certain diseases and the need to study different age groups [4]. Concerns about the protection of vulnerable patient populations, like children, who cannot usually consent for themselves [4], [9], [10], [11], [12], [13], [14], add to the complexity of recruiting to paediatric trials and continue to drive the promotion of special safeguards for children's trials [15].

Reflecting the history of low clinical trial activity in paediatrics, most research examining clinical trial recruitment has focussed on adult trials. But the complexities involved in paediatric trials mean that lessons from adult clinical trials cannot simply be extrapolated to children's trials. Despite the variety of reported challenges in recruiting children to trials, most paediatric recruitment research has focussed narrowly on improving families' recall and understanding of trials [16], [17], [18], [19]. Such research is relevant but it cannot tell us what parents and practitioners themselves consider important about how information about clinical trials is communicated and the way that trial recruitment is conducted [20]. Each party will have unique perspectives on recruitment. Research which simultaneously explores and compares their experiences is important to provide a comprehensive understanding [21] of how recruitment and its conduct may be improved. Therefore, with the aim of identifying strategies to improve recruitment and its conduct, this study compared practitioners' and parents' accounts of the invitation to enter a child into clinical trial.

To explore what was important to participants during trial recruitment we designed a qualitative study. Because our study (Processes in recruitment to randomised controlled trials of medicines for children [short study name, RECRUIT]) focussed on communication during trial recruitment we designed it to take account of a fundamental complexity in human communication: how two people experience and interpret a conversation can differ markedly from what is evident in their dialogue [22], [23], [24]. Strategies to improve the experience of clinical trial recruitment may be misguided or poorly targeted if they are informed by studies that rely on either dialogue or experience alone. For example, strategies to improve patient-practitioner communication have often been based on dialogue without taking account of experiential data; these have frequently failed to demonstrate improvements that patients or practitioners recognise [25], [26], [27], [28], [29], [30], [31]. The reverse - analysing participants' accounts of their experiences of communication without reference to their dialogue - is also likely to be limited [32]. Therefore, we collected both types of data.

## Methods

### Ethics statement

A UK National Health Service ethics committee gave approval for the study (Northwest 5 Research Ethics Committee: 07/MRE08/6). Signed informed consent was obtained from all participants.

### Study design

In this qualitative study, we audio-recorded dialogue during clinical trial recruitment discussions and interviewed participants about their experiences of these discussions. In collecting and triangulating [32] these two sorts of data we aimed to identify strategies to improve recruitment that took account of both the ‘look’ (the form of communication - what is said) and ‘feel’ (the meaning of communication - what is experienced) during clinical trial recruitment.

### Study quality

In qualitative research, data triangulation has long been recognised as important for advancing understanding [33]. In our study, data triangulation took two forms: ‘data-type’ triangulation (see previous section) and ‘informant’ triangulation whereby we compared practitioners' and parents' accounts. We describe below the other procedures [34], [35] that we used to ensure the quality and rigour of our sampling, data collection and analysis.

### Sampling of trials

RECRUIT was a ‘research on research’ study that ran alongside four placebo-controlled randomised clinical trials of medicines for children. The trials were purposively sampled. All were public or charity funded trials adopted by the National Institute for Health Research Medicines for Children Research Network (MCRN). As such, the trials addressed pressing clinical questions and avoided obvious design problems that would adversely impact on recruitment. Our decision to focus on these trials was also guided by knowing that scarce resource is at stake if such trials encounter recruitment difficulties. We selected each of the four trials to represent different conditions, disease status and trial design to maximise the transferability of findings. The trials also differed in the timing and circumstances of the approach for recruitment and in the relationship between family and the practitioners responsible for recruitment as described in Text S1. For logistical reasons, we selected sites in the North West of England where possible. Two or three teams from each trial facilitated RECRUIT.

### Sampling of families and practitioners

Each trial had an initial target to recruit a sub-sample of 15 families to RECRUIT using a mix of consecutive and purposive sampling. We used consecutive sampling to minimize ‘gatekeeper bias’ whereby, for example, practitioners might avoid approaching parents with whom they anticipated communication might be difficult [36]. We used purposive sampling to access families who had different trajectories in relation to the trials (i.e. remained on the trial, were ineligible, declined or withdrew). In particular, as we neared the target for the MENDS trial, we used purposive sampling to enrol families who declined MENDS, as our access to decliners in the wider RECRUIT sample was limited. We accessed these families after their decision to decline the trial and therefore no recorded trial discussion was available for them. Sampling of practitioners was partly tied to the sampling of families in that we selected those practitioners directly involved in the trial approach and for whom audio-recordings of trial discussions were available. However, we also purposely sampled other practitioners who were likely to have informative experiences of trial recruitment, arising from their different roles in the four trials or their experience of other trials. We sampled both doctors and research nurses, as each profession is closely involved in discussing trials with families in the UK, and included practitioners who were relatively new to clinical trial activity, as well as those with more experience.

### Recruitment

Practitioners facilitating RECRUIT routinely sought permission to audio-record trial discussions from families whom they approached for their respective trials. They described RECRUIT briefly and asked the families for permission to pass their contact details to the RECRUIT team. A member of the RECRUIT team discussed the study in full with the family and sought their consent to participate further, explaining the RECRUIT team's independence from the trial and clinical team. Audio-recordings of the trial discussions were retained by practitioners and only released to the RECRUIT team after the written consent of participants was obtained. If the family declined RECRUIT, the recorded trial discussion was erased. Where families were approached without a recorded trial discussion, the practitioner described the RECRUIT study and asked for permission to pass the family's contact details to the RECRUIT team. We sampled in parallel with analysis until the point when additional data did not alter the analysis.

### Interviews

VS and ES conducted in-depth, semi-structured interviews with parents and practitioners. Interviews were conversational and responsive to participants, thereby allowing full exploration. Interview questions were, nevertheless, informed by topic guides (see Text S2) to ensure core topics were discussed. We initially based the topic guides on our review of the literature and advice from steering group members, and developed them over the course of the study to allow exploration of topics whose importance became clear as the analysis developed. We also obtained demographic details from participants. Parents' postcodes were used to calculate Index of Multiple Deprivation (IMD) scores, which are used by UK researchers as indicators of deprivation in small geographical areas [37]. IMD scores enable ranking of deprivation in areas based on a combination of domains comprising income, education, health, housing, services and living environment [38].

### Analysis

We audio-recorded all interviews, transcribed them verbatim, then checked and anonymised the transcripts. Analysis was broadly interpretative, and followed the general principles of the constant comparative method [39], [40], cycling between the developing analysis and new data. We analysed interview transcripts for evidence of the families' experiences, needs and priorities when approached about a trial. For practitioners we analysed for evidence about their goals in discussing the trials with families and how they responded to families' cues. For trial discussions, we examined the focus of the dialogue, and the types of questions asked (attending to practitioners' invitations to elicit parents' thoughts and questions about the trial and explore their understanding) and responses given. We also calculated the proportion of speech (total utterances spoken by parent/total utterances spoken) and number of questions asked by parents as indicators of their ‘observed’ level of interactivity. This allowed us to compare the ‘look’ of the trial discussion, in terms of parents' interactivity in the dialogue, with their experience of the ‘feel’ of the discussion. We also recorded the frequency of practitioners' use of open and closed questions.

VS led the analysis with assistance from ES, and in consultation with BY to ensure investigator triangulation [32]. We read transcripts several times to compare within and between transcripts, discussing them on numerous occasions to interrogate the data and explore alternative explanations. Insights from these discussions were used to develop theoretical categories and ‘test’ the developing analysis. We organised the categories into a framework to code and index the transcripts using QSR NVivo 8 software. We extracted data relevant to these categories and the trial approach, whilst interpreting the extracts with reference to the transcript as a whole, as well as to the proximal content, before assigning the extracts to the categories and checking their assignment. We continually reviewed the theoretical categories in the light of new data, modifying these to ensure they fitted with the data whilst also accounting for deviant cases. Further investigator triangulation involved HH, RLS and PRW reviewing detailed reports of the analysis containing extensive data extracts and then using their comments to further refine the analysis. We used respondent validation, whereby we discussed our findings with practitioner and parent representatives, several of whom were members of the study steering group. We kept audit-trail records of the developing analysis, including definitions of the key theoretical categories. Finally, we examined the quality of the developing analysis according to its coherence and theoretical validity, whereby we explored links between our findings and theoretical ideas in the wider literature [32], [34], [35].

To evidence our interpretations we present some counts and percentages for the main categories [41], [42], as well as verbatim extracts from the data. For these extracts, parents' interviews are signified by identification codes beginning ‘F’, those from practitioners beginning ‘P’. For extracts from parent interviews we indicate whether the family consented, declined, was ineligible or withdrew from the trials. We signify extracts from parent-practitioner trial discussions with codes ‘TD’ (followed by parental identification numbers) and indicate in the text which party is being quoted.

## Results

### Participants

**Figure 1:**
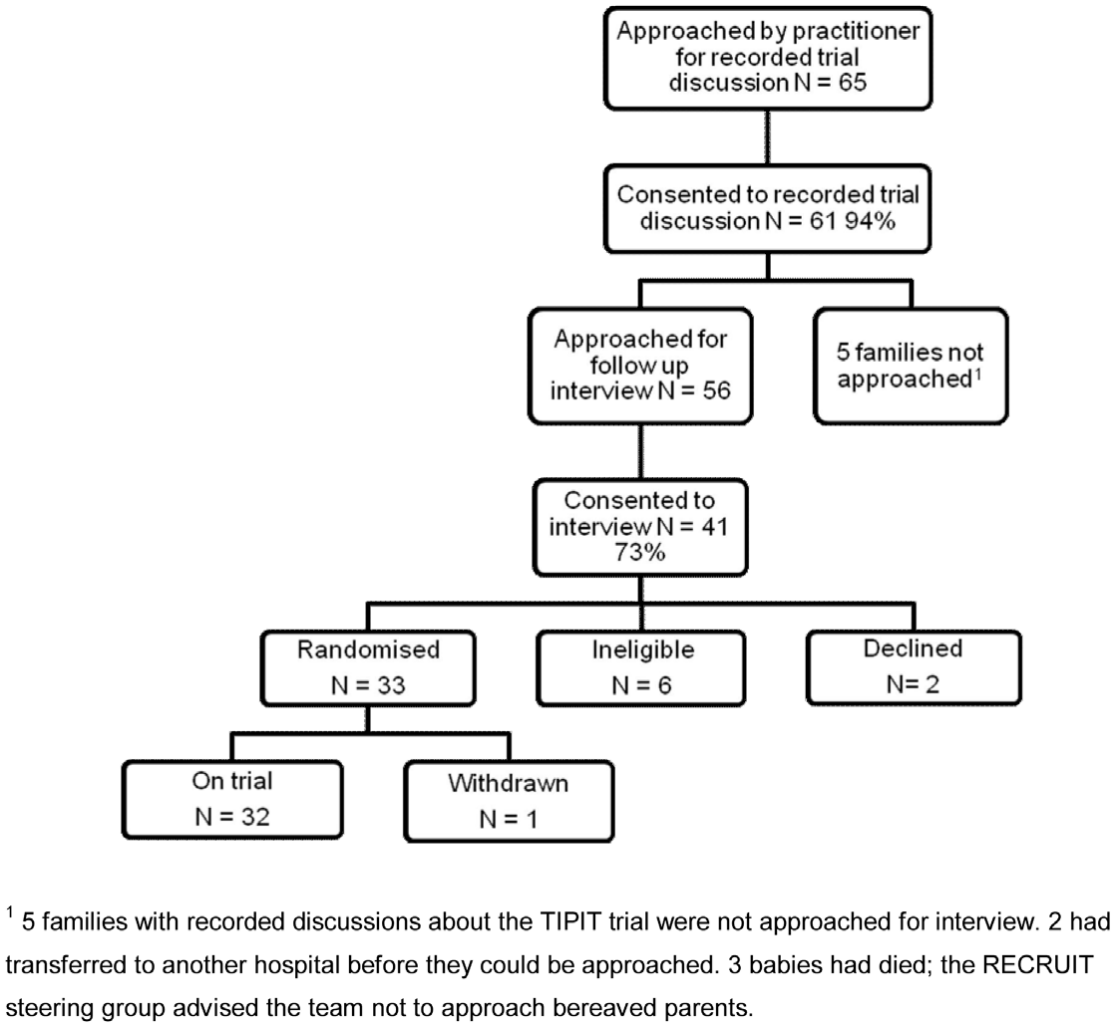
Recruitment of families with recorded trial discussion to RECRUIT. Figure pertaining to the recruitment of families to the RECRUIT study whose trial discussion had been recorded by the trial practitioner facilitating RECRUIT. The families' trajectory in relation to the trials is shown.

**Figure 2:**
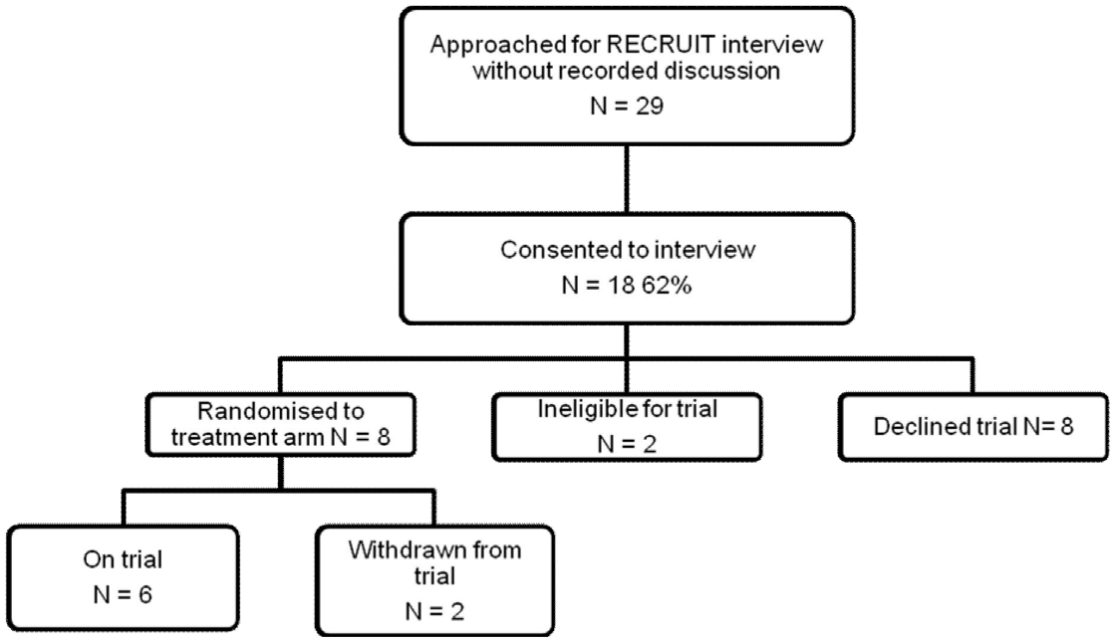
Recruitment of families approached about RECRUIT who did not have a recorded trial discussion. Figure pertaining to the recruitment of families to the RECRUIT study where there was no recorded trial discussion with a facilitating practitioner. In all cases, RECRUIT interviews were conducted after decisions about trial entry and randomisation. The families' trajectory in relation to the trial is shown.

We interviewed members of 59 families (58 mothers, 3 fathers). Figures 1 and 2 illustrate the numbers of families participating in different elements of RECRUIT by their trajectory in relation to the trials. Recorded trial discussions were available for 41 of these families, all of whom completed follow up interviews with the RECRUIT team. Interviews took place a median of 42 days after the recorded trial discussion (range 14–126) and lasted approximately 45 to 60 minutes. We interviewed 48 of the 59 families in their homes, eight at the trial site and three were telephone interviews. Of the 33 practitioners approached, we interviewed 31 (94%). 12 were research nurses who were part of the trial teams for one of the four trials, 14 were doctors who were also members of the trial teams and five were doctors who regularly recruit to trials but were not recruiting to any of the four participating trials. The latter were senior practitioners who responded to an invitation at one of the participating centres. All practitioners were interviewed face-to-face in a private room or workspace and their interviews lasted approximately 45 to 60 minutes. Interviews took place between March 2008 and January 2010. Table 1 shows parent demographics and trial participation status for each of the four trials and breakdown of practitioners by trial.

**Table 1:** Demographic and trial participation trajectory of parents participating in RECRUIT by individual trial and breakdown of practitioners by individual trial.

	MASCOT	MENDS	POP	TIPIT
N (% of total 59)	10 (17%)	22 (37%)	15 (26%)	12 (20%)
N: Randomised	5	9	13	11
Declined trial	1	8^1^	0	1
Ineligible for trial	4	4	0	0
Withdrawn from trial	0	1	2	0
Median (range) days	35	38	42	52
between recorded trial	(30–69)	(14–69)	(14–119)	(20–126)
discussion and RECRUIT interview	N = 6	N = 15	N = 8	N = 12
Median (range) age of	10	7	13	0^2^
child in years	(8–14)	(4–13)	(4–16)	
N (%) **not** self indentified as white British	0	2 (9%)	1 (6.67%)	6 (50%)
Mean (s.d.) Index of Multiple Deprivation	35.78	44.38	20.34^4^	43.78
score^3^	(25.06)	(24.22)	(16.37)	(12.20)
Doctors^5^	2	4	3	5
Research nurses	4	3	4	1

1 7 of the 8 families who declined MENDS, did so before attending a clinic appointment and as such there are no recorded trial discussions for them. In these instances, parents were interviewed about their discussions with community paediatricians, telephone conversations with the research nurse and their views on the trial and the PIL.

2 TIPIT is a neonatal trial; hence the median age is 0.

3 Higher scores indicate greater deprivation.

4 Mean and standard deviation exclude 3 families from Northern Ireland.

5 Five doctors were also interviewed who regularly recruited to trials but were not recruiting to the four participating trials.

### Interactivity in trial discussions

Parents generally said little during the trial discussions –their median proportion of speech (total utterances by parent/total utterances) was 16% (range 1–49%). In 12 discussions parents contributed 10% or less of the total dialogue, in 16, parents contributed between 11 and 24% of the dialogue and in 13 they contributed 25% or more.

The 12 discussions where parents contributed 10% or less of the dialogue were often the first formal conversation about the trial. Practitioners systematically presented key trial information in ‘chunks’, which they periodically interspersed with brief closed questions such as “*alright?*” or “*okay?*”. Parents mostly responded with a brief affirmation “*okay*” “*right*” “*yeah*” to confirm that they understood. For some parents their contributions largely comprised such minimal responses. In 6/12 discussions practitioners invited parents' comments or questions about the trial on at least one occasion e.g. “*That's a lot of information so far. Have you any questions you want to ask me?*” (TD42) but not in the remaining six. Practitioners rarely asked open questions to explore whether the parent had understood the information.

In the 13 discussions where parents contributed 25% or more of the dialogue, practitioners' questions mostly focussed on the child's condition and were aimed at establishing eligibility rather than eliciting parents' thoughts or questions about the trial or exploring their understanding (consent was taken after 10/13 discussions). In 6/13 of these ‘high interactivity’ discussions practitioners sought parents' views of the trial by asking at least one open-ended question. These were often about the Participant Information Leaflet (PIL) and occurred at the outset of the discussion “*you've had the information sheets […] what did you think of that when you, you read through it?*” (TD18). However, practitioners still tended to use closed questions to enquire about parents' understanding of the trial: “*In a nutshell, does that make sense?*” (TD3); “*Are you with me so far?*” (TD2).

Across all the groups, parents asked few questions during the recorded trial discussion (median 1, range 0–7). 10 asked no questions at all, 13 asked only one. Irrespective of the level of interactivity, practitioners always gave reassurances on trial safety and commented on the absence of trial procedures that might distress a child. In this way, practitioners may have pre-empted parents' questions, contributing to their relatively low number.

### Parents' experience of verbal and written trial information

Though parents' interactivity in the trial discussions was often low, they did not tell us that they felt inhibited or constrained in any way. On the contrary, parents often described positive experiences of the verbal information they had received, and emphasised their sense of ease in asking questions (see Box 2). Some explained that they had not asked questions during the trial discussion because they were happy with the information they had received *“It was explained the way it was meant to be explained. Anybody could have understood it”* (F2 declined) and because they felt comfortable in contacting the trial team with any questions as these occurred, “*Most doctors wouldn't say, ‘Here's my mobile. Any worries, just phone me there and then'*” (F48, ineligible) “*ring her anytime*” (F36, declined). Indeed, parents described practitioners as “*friendly*” (F56, consented) “*approachable*” (F13, consented) “*open and honest*” (F51, consented) “*relaxed*” (F60, consented), “*comfortable to talk to*” (F38, declined).

Many parents were mildly critical of the trial PILs, referring to them in ways that emphasised their length and wordiness: “*It looks like half a forest*” (F54, consented). It was not possible to determine how many parents were deterred from participating in the trials because of the length and complexity of the PILs but 14/59 parents we interviewed explicitly stated that they personally found the PIL too long or complicated. A further four commented that they thought the PILs might be too complex for other parents. A few parents pointed to information that was absent from the PILs that they believed to be important, such as the licensing of the trial medication (F33), how to take trial medication (F16), need for urine samples (F25), use of non-trial medication (F59) and eligibility criteria (F5). Despite these problems parents emphasised how valuable the PILs had been in enabling them to reflect on the trial in their own time and space. Almost without exception however parents placed greater value on the face-to-face discussion than they did on PILs and would not consider participating in a trial without a personal approach.

*If that just came through the door and I didn't feel it'd got […] any personal contact, I'd just think, ‘no, I'm not doing it’.* (F11, consented)

### Parents' experience of the trial discussions as a social encounter

When asked about the recorded trial discussion, rather than focussing on its informational content parents emphasised their experience of the discussion as a social encounter and their experiences of the practitioners. Regardless of their observed level of interactivity, parents spoke of their sense of comfort during the discussion, their strong confidence in and liking for the practitioner, and their sense that the trial was safe and that their child's health (rather than the trial) was the practitioner's overriding concern. This sense of security was sometimes linked to the knowledge that the trial was being conducted with the support of a familiar and trusted clinical team. However, there was no consistent opinion from parents on whether it was better to be approached by the child's regular practitioner or someone who was not responsible for their child's clinical care and so was not known to them. Indeed, parents tended to emphasise the benefit of whichever ‘model’ they had encountered. Parents also highly valued practitioners' consideration and the kindness and commitment of the trial team, particularly their attentiveness to the needs and preferences of their child (for quotes, see Text S3). We saw few contrasts between the accounts of parents who declined the trial and those who consented (Text S4).

### Parents' experiences of the timing of the trial approach

In two of the trials, parents were sometimes approached when they were fearful for their child's survival or well-being and it was not uncommon for these parents to acknowledge that they had found it difficult to concentrate during the trial discussion. As one mother approached about the neonatal trial commented, *“it went sort of like in one ear and out the other […] she was so small and so poorly”* (F46 consented). However, when asked if the trial discussion could have been better handled this mother echoed other parents in remarking:

*No, I don't think so. The doctor was really nice, he was clear and asked […] if I had any questions either that day or later […] to speak to them.* (F46 consented)

Although acknowledging the difficulty of discussing a clinical trial, parents in these distressing circumstances were generally accepting of the need for research and the need for the approach to be made. When asked whether she had been approached about the trial soon after the birth of her son, one mother responded “*the sooner really you get in there, it's the better isn't it?*” (F9, consented)

Two parents of children with a chronic condition felt that the timing of the trial approach could have been better “*if they'd given us even an hour for the diagnosis to sink in*” (F25, withdrawn) “*it was her first admission […] I was worried about that*” (F12, consented). However, this contrasted with one parent who viewed being approached when her child was unwell and an inpatient as appropriate because “*there were a lot more people on hand*” (F13 consented) to answer her questions. What mattered for this mother was the manner of the approach rather than its timing “*I think if they do it in the right way then it's okay to approach*” (F13 consented).

Interestingly, several parents described being “excited” (e.g. F1, F10, F21) at being asked if their child would participate and remarked on how “passionate” (F51) and “enthusiastic” (F10) practitioners were about research. This seemed to inspire parents' confidence in practitioners' expertise and commitment. A few parents described how they would have felt disappointed if they had not been invited:

*You don't want to think […] there's some sort of a trial that could improve your child's [condition] and your child hasn't been offered that […] I would like to be asked and be given the opportunity to say no.* (F50, ineligible)

Parents also emphasised how making a decision about their child at such a difficult time gave them a sense that they were involved in their child's care when there was little else they could do for their child.

*A parent needs that little bit of control, just so that they know there is still something that they're doing for their child, because other than that there is nothing.* (F29, consented)

Without exception and irrespective of interactivity in the trial discussion, parents felt that the decision on trial entry was theirs, said they were satisfied with their decision and that they would have been able to decline the trial if they had wished.

### Practitioners' experiences of communication with parents

Practitioners spoke more about the content of trial discussions and less about their experience of the process as a social encounter than parents. Practitioners were particularly concerned to ensure that parents understood the trial and described how the amount and complexity of information, particularly written information they had to give parents, undermined this objective. They commented that PILs were “*not straightforward*” (P12) “*too long*” (P2) “*too detailed, too comprehensive, too busy*” (P3) and “*too complex*” (P9). In total 24/31 commented that the leaflets were too long and complex with a further three stating that although they were happy with the trial PIL they thought that PILs in general were too complicated. Many practitioners spoke of how the requirements of ethics guidelines and committees resulted in PILs which could be “*threatening*” (P8) or “*overwhelming*” (P15) for parents and which “*turned people off*” (P1) or were not read at all. While practitioners were critical of how current recommendations shaped PILs, most acknowledged their necessity and value in communicating about trials and we observed them using PILs to open and guide the trial discussions. Like parents however, practitioners valued the face-to-face discussion more highly than the PILs (Text S5) particularly because face-to-face communication enabled them to gauge how information was received by parents and respond to their cues. Practitioners spoke of their concern when families were eager to agree to the trial when practitioners did not feel that families had sufficiently understood the trial.

*The families I worry about […] are the families that just say, […] ‘it's all right, I don't need to read the information sheet. I'm happy, whatever you say’*. (P5)

Practitioners described how they felt “*happier*” (P6) when families had *“got questions because you feel like they're wanting to be fully informed themselves”* (P6) and commented on how, in the absence of such feedback, *“it was hard to know whether or not he truly understood what he was consenting to”* (P28).

Occasionally practitioners remarked on discussions where they had annoyed or deterred a parent by continuing to explain the trial after the parent had said they were happy for their child to enter the trial “*the fact that they've said ‘yes I want to be part of the study’ and you somehow want to argue with them*” (P1); “*he seemed to be getting […] more annoyed with me the more I was talking*” (P28). This posed a dilemma for practitioners in making sure parents understood what they are consenting to, whilst also ensuring that the parent felt listened to. Other practitioners emphasised how parents' understanding was something that was achieved over time and might require several discussions. However, some questioned whether informed consent was achievable at all, particularly when the child's condition was critical. They referred to how some parents could make a decision that they (the parents) were comfortable with, based on their attitudes to research and on the most essential information about the trial. They regarded such families' decisions not to seek detailed information about the trial as an appropriate form of autonomy in a stressful situation (Box 4).

A number of practitioners made a clear distinction between their own particular approach and the ‘required’ content of the trial discussion as conveyed in programmes such as Good Clinical Practice (GCP) training “*Oh GCP, but that […] doesn't teach you how to talk to people. It just tells you what the rules are really*” (P28).

These practitioners emphasised the need to be adaptable to the needs of individual parents.

*Giving tips implies that there's a right and a wrong way of doing it and I'm not sure that there is, except to be sensitive to different people having different needs at different times and to listen.* (P11)

### Practitioners' comfort with approaching families

Practitioners described different levels and sources of difficulty in approaching families about trials. The majority did not describe approaching families as something that they found personally difficult, but many nevertheless believed that being approached could exacerbate the emotional impact of the child's illness “*these are very, very sick kids […] you're going up to them and this is yet another consideration for them*” (P2). Such concerns were particularly prominent in the rheumatology and neonatal trials where the children were often severely or critically ill. Other practitioners expressed an intense sense of personal disquiet or anxiety about approaching families about trials *“each parent is different and causes me great anxiety”* (P16), *“I will go and approach them but I feel, I feel very uncomfortable doing it every single time”* (P18). The primary source of these practitioners' discomfort was the intensity of families' fears and distress. This led some to ask searching questions about the morality of approaching such families “*this family's at a terrible time and really is it right to be asking them to do this?*” (P19). Several research nurses expressed a lack of confidence when working on trials outside of their specialty and individual practitioners spoke in strong terms of a range of other difficulties, such as the pressure to reach recruitment targets (P26), feeling “*like a salesman*” (P19), discomfort with children's medication being selected at random (P31), and concerns that families' vulnerability meant they were liable to be unduly influenced by practitioners (P18). A few practitioners described how their belief in the importance of research helped to ease their own discomfort.

*Much more stressful for the family and much more stressful for you […] it's only because you believe that intervention is critically important to investigate that you feel that you can kind of carry on* (P12)

Occasionally, however practitioners echoed the sentiments of the parents saying that on the whole, parents did not object to being approached about research:

*The more you recruit people […] you feel less apprehensive yourself about asking […] you realise that actually asking them and them saying ‘no’, you haven't upset them. You […] haven't changed anything.* (P28)

## Discussion

This study has highlighted a striking disparity between parents' willingness to be approached about their children's participation in clinical trials and practitioners' discomfort and awkwardness about recruiting children to the trials. Whether they consented or declined, no parent objected to being approached about any of the clinical trials and many described the way the approach was conducted in highly positive terms. Even in the most difficult circumstances, parents told us that they understood and accepted why they were asked and the timing of the approach, providing it was made in a considerate way. Some even described feeling excited at being approached about the trial, spoke of how they valued practitioners' passion for research, or stated that they would have been disappointed had they not been asked. Practitioners did not describe approaching families about trials in these terms; many regarded trials as an unwelcome burden for families and some felt personally uncomfortable about approaching families.

Meaning arises in how people experience what is said [43] and it cannot simply be ‘observed’ in their dialogue [44]. This study used a novel, multi-perspective design to investigate recruitment in a way that took account of this fundamental complexity in communication. It is one of the few studies of trial communication to do this, accessing its ‘look’ and ‘feel’, and the only study to do so from the perspectives of both parents and practitioners. Previous research on the ‘look’ of trial communication has been critical of how practitioners communicate about trials and encouraged them to facilitate interactivity [18], [45], [46], [47], [48], [49]. Interactivity may be important to facilitate patient understanding of trials [47] but the ‘feel’ of trial communication to both parties cannot be neglected. In this study parents were positive about practitioners' communication despite their low interaction in the trial discussions. Meanwhile, practitioners described how they sometimes felt obliged to constrain their explanations to show parents that they were listening to them. Irrespective of their interactivity parents felt able to contribute to the discussions and had a sense of ownership of and satisfaction with their decisions. Therefore, while parents wanted caring and expert practitioners to explain the trials to them, they did not necessarily want to actively contribute to the discussions.

A few families were approached about the trials by their child's doctors, although most were approached by practitioners who were not responsible for the child's clinical care. This may have impacted on parental interactivity – parents may have interacted more if the trial discussions had been initiated by practitioners who were known to parents and/or could answer questions about their child's clinical care. Future research is necessary to investigate what functions parental interactivity serves during trial discussions and how it is influenced by the relationship parents have with the recruiting practitioner and his/her role in the child's clinical care. Similarly, further research would be required to investigate how practitioners' comfort in approaching families varies depending on their relationship with the family. Our interviews with parents and practitioners indicated that neither had a consistent preference, rather they were happy with whichever ‘model’ they had experienced regardless of whether this involved discussing the trial within or outside an existing clinical relationship [50].

Our inclusion of trials that recruited children who were severely or critically ill has confirmed that recruitment to such trials is more challenging for both parties. However, even in these circumstances, parents did not construe the trial approach as an unwelcome burden. Other studies have shown that parents are comfortable with the consent process as a whole [51] and value their participation in it [52]. Evidence on adult patients' routine clinical care has indicated that not all patients want an active role in discussions with practitioners [53]. More fundamentally, evidence also indicates that interactivity is not synonymous with a sense of involvement, and that it is the opportunity to be involved, not necessarily its enactment that patients value [22], [54], [55], together with the sense of being free to accept or decline the practitioner's suggestion [44]. Adult patients with cancer and their families stressed the importance of practitioner communication behaviour that balances the information content of the discussion with reflective, supportive and responsive behaviour to support decision making [56], [57]. Our findings offer evidence that the same is true of parents considering trial entry for their child.

### Limitations

This study had some limitations. All practitioners interviewed were actively engaged in research and only 13/59 families declined or withdrew from the trials. The divergences that we found between parents and practitioners could be a reflection of the relatively small number of declining families in our sample. However, we identified few differences between parents who joined the trials and those who declined. A further question is whether parents were reluctant to directly criticise practitioners because they were worried that their relationships with practitioners or their child's clinical care might be affected. We think this is unlikely for two reasons; firstly we were careful to explain to families our independence from the trial teams, and secondly a reluctance to criticise might lead to neutral or mildly positive accounts rather than the highly positive ones that we observed. Nevertheless, further research with parents who decline or withdraw their children from clinical trials is needed.

The time lag between the recorded trial discussions and the interviews may have led to some details of individual trial discussions being forgotten. But our aim was to explore participants' experiences of the trial approach rather than to test what they could recall of it. We think that those aspects of the trial approach which participants focussed upon in their interviews are likely to be indicative of what they considered important during trial recruitment. Also, we cannot entirely rule out the possibility that the audio-recording influenced the trial discussions. However, both audio- and video-recordings have been extensively used in studies of clinical communication [58], [59], [60] and practitioners and patients seem to rapidly habituate to such recording so that it has little influence on their communication [61]. In any event, 18 parents had not participated in audio-recorded trial discussions and we found no evidence that experiences of the trial approach differed, in ways that could be attributed to the audio-recording, between those parents who had participated in audio-recorded trial discussions and those who had not.

Mothers predominated in our sample of parents, which included only three fathers, two of whom were interviewed jointly with the child's mother. This reflects parenting norms in UK society, as well as the relative absence of fathers from the trial discussions. We were unable to explore the effect of practitioner gender, which was confounded by profession as all 12 of the nurses were female while the majority of doctors were male.

### Implications for practice

While we used many procedures to ensure this study's rigour, quality in qualitative research requires more than procedural rigour and should also be judged by the insights a study offers to practice [62], [63]. Divergence between the ‘look’ and ‘feel’ of trial recruitment led us to focus on the social aspects of trial discussions and the implications for recruitment practice. Previous research and most recruitment training has highlighted the importance of procedures aimed at ensuring informed consent, yet our study indicated how the parent-practitioner relationship and the everyday norms of clinical care had a crucial role in parents' experience of their decision. Informed consent procedures are important, but rigidity in their implementation could interfere with parents' sense of involvement and ownership. Parents emphasised how an environment in which they felt valued and comfortable to interject was key to how they viewed their decisions. Many practitioners will recognise the importance of communicating in ways that foster the right interactional environment for individual patients [64]. Given the importance that parents place on the social milieu, it would be a mistake if recruitment training obstructed practitioners' discretion and focussed exclusively on the procedural aspects of informed consent at the expense of its social norms.

Practitioners were far from complacent about approaching families about trials. Perhaps linked to their perceptions of children's and families' vulnerability they were particularly concerned to avoid burdening families. While this conscientiousness is broadly reassuring, approaching families seemed arduous and aversive for some practitioners. Previous research has focussed on improving patients' experience of recruitment, but our findings suggest that diverting some of this effort to improving practitioners' experiences of recruitment is also important. Our findings are directly relevant to the children's clinical community. But they also speak to a wider constituency of practitioners – those who recruit to trials involving other patient groups perceived as vulnerable, such as trials in emergency medicine and dementia. If practitioners find recruiting such patients to trials aversive, some will almost certainly be inclined to avoid approaching eligible patients. If eligible patients are not approached it is damaging for research, [10], [11], [65] and runs counter to the emphasis on distributive justice in recent guidelines on research conduct [15], [66], [67]. Practitioners' experiences of recruiting to trials could also leave them demoralised. We suggest that there is a need for ‘moral support’ or mentoring for recruiting practitioners and that this should include education about how families perceive being invited to enter their children into clinical trials. This may be particularly beneficial for less experienced practitioners and those working in specialties where patients and their families are perceived to be particularly vulnerable.

Finally, parents and practitioners were in agreement that PILs were too long and complicated and both groups would like to see these documents be made more reader-friendly. These findings add to the growing body of literature indicating that regulatory guidelines are leading to PILs that are at odds with the requirements of families and practitioners and may even be damaging to families' understanding [68] [69].

## Supporting Information

Text S1 **Participating trials and the recruitment process**
(DOC)

Text S2 **Summary topic guides for interviews**
(DOC)

Text S3 **Consenting parent's views on the verbal and written information they received**
(DOC)

Text S4 **The experiences of parents who declined**
(DOC)

Text S5 **Practitioners' views on verbal and written information for families**
(DOC)
