# Supplementary material for: Communication about Children's Clinical Trials as Observed and Experienced: Qualitative Study of Parents and Practitioners
Source: PLoS One. 2011 Jul 12;6(7):e21604. doi: 10.1371/journal.pone.0021604 (PMC3134466; doi:10.1371/journal.pone.0021604)
Supplement: Text S1 — Participating trials and the recruitment process (DOC) [file pone.0021604.s001.doc]

**Participating trials and the recruitment process**

**MASCOT – *Management of asthma in school age children on therapy***

**Summary:** Double-blinded RCT comparing the add-on treatments salmeterol (long acting beta_2_ agonist) and montelukast (leukotriene receptor antagonist) for young people whose asthma was poorly controlled with fluticasone (low-dose inhaled corticosteroid).

**Population:** 6 – 15 year olds whose asthma was poorly controlled with fluticasone.

**Trial arms:** i) Inhaled fluticasone + placebo tablet; ii) inhaled fluticasone and salmeterol (combined inhaler) + placebo tablet; iii) inhaled fluticasone + montelukast tablet.

**Treatment period:** Four week run-in period during which families were provided with information about asthma and its management, followed by a 48 week treatment phase.

**Recruitment process:** Initial approach usually via letter from the GP or by a doctor when the child was attending a secondary care centre. Interested families received Participant Information Leaflets (PILs) and a telephone call from a research nurse before attending an appointment specifically arranged to discuss trial entry.

**MENDS – *The use of melatonin in children with neurodevelopmental disorders and impaired sleep***

**Summary:** Double-blinded RCT of melatonin in young people aged 3-15 years with neurodevelopmental disorders and impaired sleep.

**Population:** 3-15 year olds with neurodevelopmental disorders and impaired sleep.

**Trial arms:** i) Melatonin; ii) placebo.

**Treatment period:** 4 week behavioural intervention (sleep management techniques) followed by 12 week treatment period.

**Recruitment process:** Parents were usually told about MENDs by their community paediatrician at a routine clinic visit. They received PILs by post and had a telephone conversation with a research nurse before attending an appointment with the nurse and paediatric neurologist specifically arranged to discuss trial entry.

**POP – *Prevention and treatment of steroid-induced osteopaenia in children and adolescents with rheumatic diseases***

**Summary:** Double-blinded RCT to investigate if the bisphosphonate risedronate or the Vit D analogue 1-alphaphydroxycholecalciferol were better than placebo in preventing/reducing bone loss in young people with rheumatic diseases treated with corticosteroids.

**Population:** 4-18 year olds with rheumatic diseases treated with corticosteroids

**Trial arms:** i) Risedronate (2n children); ii) one alpha (2n children); iii) placebo (2n children, n children received risedronate placebo, n received one alpha placebo). All groups received calcium and vitamin D supplementation daily.

**Treatment period:** 52 weeks

**Recruitment process:** POP was usually briefly introduced to families by a member of the clinical team responsible for the child’s care. POP’s design allowed considerable flexibility so practitioners could select an appropriate time to approach the family. After the initial introduction a more in-depth discussion was arranged to coincide with a routine hospital visit.

**TIPIT - *A randomised controlled trial of thyroxine in pre-term infants under 28 weeks’ gestation***

**Summary:** Double-blinded RCT of thyroid hormone supplementation in babies born under 28 weeks’ gestation.

**Population:** Pre-term infants under 28 weeks’ gestation

**Trial arms:** i) Thyroxine; ii) placebo

**Treatment period:** Trial medication commenced within 5 days of birth and was given every day, through an IV or feeding tube, until the baby was 32 weeks, with follow-up until the baby went home.

**Recruitment process:** TIPIT was initially introduced to parents by a practitioner, usually on the neonatal unit or at the mother’s bedside and frequently by a practitioner who was not otherwise involved in a baby’s care.
