# Supplementary material for: Communication about Children's Clinical Trials as Observed and Experienced: Qualitative Study of Parents and Practitioners
Source: PLoS One. 2011 Jul 12;6(7):e21604. doi: 10.1371/journal.pone.0021604 (PMC3134466; doi:10.1371/journal.pone.0021604)
Supplement: Text S2 — Summary topic guides for interviews (DOC) [file pone.0021604.s002.doc]

**S1: Summary topic guides for interviews**

**Summary topic guide for interviewing parents**

Interviews with parents included eight broad topics each with a number of subtopics. The main topics are listed below with examples of subtopics. These are not exhaustive.

**General family background:** Including impact of condition on child’s and family’s life and how they came to be discussing participation in a trial.

**General introduction to trial:** Including discussion of what “randomised controlled trial” meant to the parent and how they felt about their child being asked to participate in a trial.

**Recorded trial discussion:** Specifically about the recorded trial discussion with the practitioner including what was explained about the trial; what was clear and unclear; their relationship with the practitioner, how they found them on that day and how they felt the discussion went.

**Trial design:** Including the parent’s own description of what the trial involved and why it was being done; discussion of randomisation and blinding; whether they had questions about the trial and how they were dealt with; any positive or negative aspects they considered about participation

**The child:** All parents were asked how their child’s needs had been considered during the trial approach. Where appropriate, there was also discussion of the child’s views on the trial and the decision that was made about trial entry.

**Decision making:** Including how the family went about making the decision; what and who influenced them most; whether they felt under pressure or able to say no; who they discussed trial participation with; how they felt about the amount of information they received, both written and in discussion with staff.

**The information sheet:** Including their views of the information sheet and whether it could be improved; whether there was sufficient time to read the information leaflet and whether they used it in their decision making.

**Views on research:** Including discussion of their familiarity with and views on medical research in general and specifically with children.

**Summary topic guide for interviewing practitioners**

Interviews with practitioners included seven broad topics each with a number of subtopics. The main topics are listed below with examples of subtopics. These are not exhaustive.

**Background information:** Including level of involvement in relation to the participating trial and with research in general.

**General experience of approaching families:** Including whether they feel comfortable approaching families about research and how they feel about approaching families if there is an existing relationship.

**Discussing the trial:** Including the different roles played by different members of the team; what topics are critical to address in a trial discussion; what are the easier and more difficult things to explain; explaining uncertainties such as randomisation and blinding and managing treatment preference.

**The families:** Including the tailoring of information; different characteristics that make it easier or more difficult to talk to families about trials; the types of questions families ask and how to manage them; the impact of the child on the trial discussion and how to involve them in the discussion; the timing of the trial discussion and the time available for decision making.

**Trial participation:** Including reticence about approaching some families; concerns about families’ not understanding the trial and how to manage that; experiences of families declining trial entry and responding to cues that a family does not want to participate.

**Information and ethics:** Including views on the PILs; views on families’ participation for altruistic reasons; whether research might be promoted more publicly; ways to make research easier whilst still retaining safeguards.

**The Practitioner:** Including motivators and demotivators for being involved in research; views on the dual role of doctor/nurse and trial recruiter; specific training on trial recruitment and development of own approach; differences between own and others’ approaches.
