# Supplementary material for: Communication about Children's Clinical Trials as Observed and Experienced: Qualitative Study of Parents and Practitioners
Source: PLoS One. 2011 Jul 12;6(7):e21604. doi: 10.1371/journal.pone.0021604 (PMC3134466; doi:10.1371/journal.pone.0021604)
Supplement: Text S3 — Consenting parent's views on the verbal and written information they received (DOC) [file pone.0021604.s003.doc]

**Consenting parent’s views on the verbal and written information they received**

**Parents were happy with the information given to them by the trial team and felt comfortable that they could approach them with any future questions:**

*They've explained everything step by step all the way [...] There's not really been a need to sort of like ask questions. The information's been there, really.* (F18)

*Especially because she was like approachable and everything [...] I didn’t feel worried about asking.* (F10)

**Parents valued the information leaflets highly however they felt that the face-to-face discussions were more important and that leaflets were long and wordy:**

*It was explained so well [...] it’s easy enough reading it on paper [...] but when somebody’s actually speaking it through it makes more sense* (F15)

*I can’t remember how many pages it was but I can remember thinking “oh my goodness”* (F8)

**Parents focussed on how the practitioner made them feel rather than the content of the conversation in describing the trial discussion:**

*They were lovely people, they were really, really nice and made us feel really welcome and really comfortable and they did explain, you know, things really, really well.* (F1)

*It weren't like a job to do it, she actually seemed interested in it. So I was happy with that [...] [Like that] she kind of cared.* (F9)

*You could see he was passionate about the, about the research and the trial, so [...] that just, just aids things. It makes it more comfortable* (F51)

*It was the way that they treated [my child] that influenced me a lot, that [...] his opinions did matter.* (F13)

*I don’t understand a lot of the medical terms and things but I know it’s not harming him or anything so for me, you know, I was like “Go ahead with it 100%”* (F41)
