# Supplementary material for: Communication about Children's Clinical Trials as Observed and Experienced: Qualitative Study of Parents and Practitioners
Source: PLoS One. 2011 Jul 12;6(7):e21604. doi: 10.1371/journal.pone.0021604 (PMC3134466; doi:10.1371/journal.pone.0021604)
Supplement: Text S4 — The experiences of parents who declined (DOC) [file pone.0021604.s004.doc]

**The experiences of parents who declined**

**Parents who declined (N=10) were positive about their experiences of the trial and the majority were indistinguishable from those who consented:**

It was not unusual for these families to also describe the practitioner as “*really friendly*” (F36) or “*absolutely lovely*” (F23)

Nor did these families report feeling pressurised by the trial practitioner to take part:

“*We knew we wouldn’t be pushed into something*” (F24)

“*Everything in terms of mannerisms, conduct, behaviour was absolutely spot on*” (F52)

However, in two cases, the parents voiced criticisms of the approach:

“*He was fine [...] he just explained the thing*” and later “*No I didn’t feel pushed, but I think the place was wrong*” (F34) (parent was approached on a busy ward)

“*He was alright. It was just the fact that I got annoyed with all the waiting and when I tried to explain to him about the [medication] he wasn’t listening at first*” (F2)

In both cases the parent had strong reasons for declining; one as a result of a misunderstanding of the practitioner’s explanation of a trial procedure and the potential for distress to her child, while the other believed that her child had had an adverse drug reaction to the medication that was being investigated in the trial; some years previously the child had been prescribed this same medication outside of the trial.
