# Supplementary material for: Communication about Children's Clinical Trials as Observed and Experienced: Qualitative Study of Parents and Practitioners
Source: PLoS One. 2011 Jul 12;6(7):e21604. doi: 10.1371/journal.pone.0021604 (PMC3134466; doi:10.1371/journal.pone.0021604)
Supplement: Text S5 — Practitioners' views on verbal and written information for families (DOC) [file pone.0021604.s005.doc]

**Practitioners’ views on verbal and written information for families**

*The face-to-face discussion is the time to make sure they really understand [...] Because it’s only on that face-to-face interaction you can look at people and you know whether they’re getting it or not (P5)*

*In fact some parents just say, ‘I wish we didn’t have all this information. I wish you’d just, go and do it’ (P7)*

*There are some that are very obviously want to know very little, and they’ll say, ‘Yes doc, go ahead, let’s do it.’ And others will ask questions. And if they ask questions I allow the time to answer them. But I don’t think it’s the same for every patient at all. I don’t tailor; I go by what those families want.* (P30)

*To really give informed consent just how much would you have to do [...] ? Would you have to go to medical school and learn all about these treatments? (P18)*

*I think it's taken a number of years before people are getting towards striking a right balance between providing crucial information, but not overwhelming the patients, overloading the, the families [...] so they can't sometimes see the wood for the trees. (P15)*

*If you do it according to the book then [the PIL] is an intimidating document which doesn’t enhance patients’ autonomy and does occasionally turn them off being involved in the trial. (P1)*

*I'd love to know though how many people actually read these information sheets. I'm not convinced that a lot of people do. (P19)*
